# Caveolin-1 and Caveolin-2 Can Be Antagonistic Partners in Inflammation and Beyond

**DOI:** 10.3389/fimmu.2017.01530

**Published:** 2017-11-17

**Authors:** Cecília Jacques Gonçalves de Almeida

**Affiliations:** ^1^Laboratório de Imunofarmacologia, Instituto Oswaldo Cruz, Fundação Oswaldo Cruz, Rio de Janeiro, Brazil

**Keywords:** caveolin, caveolae, inflammation, infection, fibrosis, proliferation, endocytosis, signaling

## Abstract

Caveolins, encoded by the CAV gene family, are the main protein components of caveolae. In most tissues, caveolin-1 (Cav-1) and caveolin-2 (Cav-2) are co-expressed, and Cav-2 targeting to caveolae depends on the formation of heterooligomers with Cav-1. Notwithstanding, Cav-2 has unpredictable activities, opposing Cav-1 in the regulation of some cellular processes. While the major roles of Cav-1 as a modulator of cell signaling in inflammatory processes and in immune responses have been extensively discussed elsewhere, the aim of this review is to focus on data revealing the distinct activity of Cav-1 and Cav-2, which suggest that these proteins act antagonistically to fine-tune a variety of cellular processes relevant to inflammation.

## Introduction

Caveolae are vesicles of 50–100 nm that form Ω-shaped invaginations when attached to the plasma membrane. Caveolins constitute a family of three structural proteins of caveolae. Caveolin-1 (Cav-1) and caveolin-2 (Cav-2) are expressed ubiquitously, except in striated muscle cells, where caveolin-3 (Cav-3) is predominant. Human Cav-3 and human Cav-1 share 65% identity and 85% similarity and display similar activities, such as the capacity to form caveolae and to regulate the activity of various proteins. Cav-1 and Cav-3 interact with many proteins through a homologous domain, named caveolin-scaffolding domain (CSD). CSD binds to caveolin-binding motifs (CBD), conserved aromatic-rich motifs present in a variety of proteins (ΦXΦXXXXΦ, ΦXXXXΦXXΦ, ΦXΦXXXXΦXXΦ, where Φ is an aromatic residue and X is any amino acid) (1). CBD is frequently found in catalytic sites, and binding to CSD usually implies inhibition of bound proteins (e.g., eNOS, EGFR, PKA, Src kinases) (2). Nevertheless, activation has also been described (e.g., insulin receptor) (3). CSD is supposed to act as scaffolds, segregating proteins in caveolae and favoring signal transduction (2), although this was recently questioned (4, 5). The corresponding region of Cav-2 is more divergent (human Cav-2 and human Cav-1 share 38% identity and 58% similarity) and does not display the property of regulation of other proteins activities.

Caveolin-1 and Cav-2 are synthesized in the endoplasmic reticulum and form stable heterooligomers of ~14–16 subunits (6). These oligomers are transported to the Golgi complex, where they interact with other caveolin oligomers and cholesterol, forming large complexes that traffic to the plasma membrane. Cavins are also required for the formation of caveolae, and other proteins are recruited to these complexes (7). Whereas Cav-1 is able to form homooligomers (8, 9), in the absence of Cav-1, Cav-2 forms monomers and dimers that localize to the Golgi complex, becoming a target for degradation. Co-expression with Cav-1 stabilizes and redistributes Cav-2 to caveolae membranes (10–12). Cav-1-deficient mice (Cav-1^−/−^) express negligible amounts of Cav-2 (circa 5%) (13, 14) and in the absence of Cav-2, Cav-1 is expressed in lower levels (around 50% in the heart and the lungs) (9).

**Figure 1:**
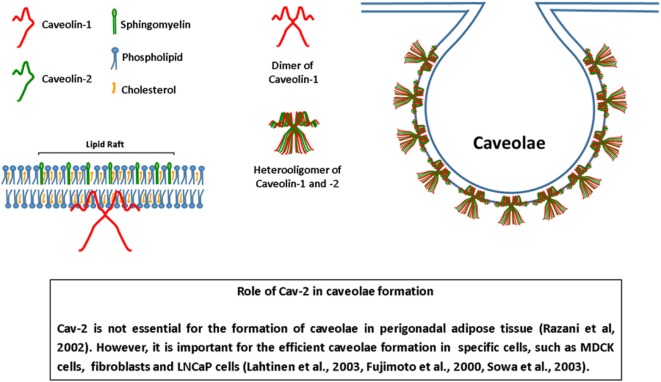
Structure of caveolae. Caveolae are invaginations of the plasma membrane, rich in sphingomyelin and cholesterol. Caveolins are the main protein components of caveolae. Caveolin-1 form homo- or heterooligomers with caveolin-2 (Cav-2) and is essential for the formation of caveolae. The role of Cav-2 in the formation of caveolae is controversial. Some cells of Cav-2^−/−^ mice form caveolae. On the other hand, evidence show that Cav-2 is necessary for the formation of caveolae in various cells. Moreover, the presence of Cav-2 can modify the morphology of caveolae, turning it deeper or attached to the plasma membrane. Adapted from Ref. (21).

While Cav-1 and Cav-3 are essential to form caveolae in non-muscle cells and muscle cells, respectively (13–15), the need for Cav-2 to form caveolae is controversial (Figure 1). For example, it has been reported that perigonadal adipocytes and lung endothelial cells of Cav-2-deficient (Cav-2^−/−^) mice still form caveolae (9). However, other studies do report that Cav-2 is essential for caveolae formation. In MDCK cells, caveolae are present in the basolateral membrane, where Cav-1 and Cav-2 form large heterooligomers. Cav-1, but not Cav-2, is present in the apical membrane, which lacks caveolae. The exogenous expression of a Cav-3 mutant prevents the formation of Cav-1-Cav-2 heterooligomers and eliminates caveolae from the basolateral membrane. Additionally, overexpression of Cav-2 increases caveolae number in the basolateral membrane (16). In fibroblasts, Cav-1 is able to form caveolae alone, but co-expression with Cav-2 leads to the formation of deeper caveolae (17). LNCaP cells, that do not express either Cav-1 or Cav-2, form plasmalemmal attached caveolae only when both proteins are exogenously expressed. In addition, this process is dependent on phosphorylation of Cav-2 Ser 23 and Ser36 (18). Besides serines, Cav-2 Tyr19 and Tyr27 are also targets for phosphorylation, which seems to be important to generate docking sites for the SH2 domain-containing proteins (19, 20). Taken together, these results suggest that not only Cav-2 is a partner of Cav-1 in the formation of caveolae, but also, contributes to signal transduction generated in these cellular platforms. Interestingly, despite cooperation and tightly associated expression, independent studies show evidence that Cav-1 and Cav-2 may exhibit opposite effects on distinct cellular processes (Table 1). Hence, it is possible that Cav-2 together with Cav-1 is involved in the fine-tuning of basic cellular processes, acting sometimes in a counterbalancing way. This would be of great relevance considering the wide range of cellular events modulated by Cav-1 such as proliferation, lipid metabolism, and cellular trafficking. The aim of this review is to bring together results from recent studies that indicate that Cav-1 and Cav-2 show antagonistic activities in various contexts, particularly in inflammation.

**Table 1:** Take-home messages.

Cav-1 and Cav-2 are tightly co-expressed and co-localize in caveolae, although they may also occur in distinct subcellular locations, and distribute differently in distinct cell types Cav-1 and Cav-2 are partners in caveolae formation Cav-1 and Cav-2 may also act in counterbalancing ways on various processes, such as angiogenesis, endocytosis, and regulation of inflammatory responses

## Cav-1 Activities in Inflammation

Inflammation is a response to noxious and damaging stimuli, such as infections, trauma, and injury, in an attempt to restore homeostasis. The inflammatory response involves a sequence of well-orchestrated events that begins with the recognition of molecules that indicate the presence of pathogens and/or tissue damage. The role of caveolins in many aspects of the inflammatory response, such as angiogenesis, leukocyte recruitment, pathogen invasion, production of inflammatory mediators, and fibrosis has been extensively demonstrated in the literature. Below are summarized the main findings on Cav-1 involvement in inflammation. For instance, it has been demonstrated that Cav-1 binds to toll-like receptors (TLRs), such as TLR4 and TLR5 (22–24). Binding of lipopolysaccharide (LPS) to TLR4 culminates with the production of inflammatory mediators, such as pro-inflammatory cytokines and nitric oxide (NO). In macrophages, the association of Cav-1 to TLR4 diminishes the production of pro-inflammatory cytokines and enhances the production of anti-inflammatory cytokines, at least in part, in a p38-dependent manner (25). Upon TLR4 activation, the ubiquitin ligase ZNRF1 ubiquitinates Cav-1 leading to its degradation, resulting in increased production of pro-inflammatory cytokines and inhibition of anti-inflammatory ones (26). TLR4 activation by LPS also stimulates heme oxygenase-1 producing carbon oxide (CO), which has known anti-inflammatory activity. CO, in turn, stimulates Cav-1–TLR4 interaction, also contributing to the feedback loop that restricts the pro-inflammatory response (24). Curiously, in endothelium, the interaction of Cav-1 with TLR4 has an opposite outcome, leading to the activation of NF-κB and the production of pro-inflammatory cytokines (22). Cav-1 activity, in this case, depends on phosphorylation of Tyr14. Cav-1 also regulates endothelium permeability as Tiruppathi and coworkers showed that LPS stimulates Cav-1 expression, suggesting that it contributes to the increase of caveolae on the endothelium cell surface and transendothelial albumin permeability (27). Moreover, Cav-1 binds to and holds eNOS inactive. In the Cav-1 absence, eNOS is hyperactive, and eNOS-derived NO nitrates IRAK4 (28) and inhibits NF-κB activation (29), diminishing the expression of pro-inflammatory genes and vascular permeability. Cav-1 is also determinant for H_2_O_2_-induced pulmonary vascular albumin hyperpermeability and hypoxic trophoblast HMGB1-induced hyperpermeability (30, 31). Endothelial Cav-1 may determine the route of neutrophil and T lymphoblasts migration through endothelial cells, although contrasting results indicate that the expression level of Cav-1 may help or hamper diapedesis depending on the cell type studied (32, 33).

After LPS-induced endotoxemia, the lungs of Cav-1^−/−^ mice exhibit less neutrophil sequestration and edema formation (29). Further, Hu and collaborators showed that Cav-1 expression in polymorphonuclear cells determines the efficiency of adhesion and chemotaxis *in vitro* and that Cav-1^−/−^ PMNs are recruited less efficiently than wildtype PMNs after perfusion with fMLP and PAF, causing fewer septa thickening and edema in mouse lungs *in vivo*. The absence of Cav-1 in PMNs is also associated with the diminished production of superoxide after stimulation with fMLP and PMA, and disruption of rafts reduce superoxide production in wildtype PMNs (34). Thus, Cav-1 in endothelial cells has pro-inflammatory activity, opposing to its anti-inflammatory activity in macrophages. Cav-1 expression in macrophages also favors the ability to phagocytose both *E. coli* and apoptotic cells *in vitro* (35, 36). Further, Cav-1^−/−^ macrophages are less effective in killing *E. coli* both *in vivo* and *in vitro*. These results correlate with diminished production of NO (36). This result contrasts with the enhanced production of NO in Cav-1^−/−^ macrophages infected by *Salmonella* (37). Interestingly, some pathogens exploit caveolae as a route of internalization that would allow their survival, since it avoids the lysosomal pathway [for reviews, see Ref. (38, 39)]. Finally, Cav-1 is also involved in the regulation of fibrosis, indicating a potential role in the isolation of pathogens and tissue repair (40–43).

The role of Cav-2 is far less studied than that of Cav1. Surprisingly, Cav-2 often shows an opposite activity compared to Cav-1. In this review, a collection of data will be presented to support this statement.

### Endothelial Proliferation/Angiogenesis

Angiogenesis is a tightly regulated process that, in adults, mainly occurs in pathological conditions, such as cancer, diabetic retinopathy, and chronic inflammation. The formation of new blood vessels involves activation of endothelial cells, vasodilation, and extracellular matrix (ECM) degradation followed by proliferation of endothelial cells that then migrate and differentiate. Evidence exists for a function of Cav-1 as a negative regulator of cell proliferation: (i) quiescent and terminally differentiated cells, such as adipocytes, endothelial cells, and type I pneumocytes contain high levels of Cav-1 (44), (ii) growth factors are able to inhibit Cav-1 expression (45), and (iii) Cav-1 inhibits cell cycle progression (46, 47). In endothelial cells, the Cav-1 expression is low during proliferation, progressively increases during differentiation, and reaches its peak just before microtubule formation. Angiogenic growth factors, such as VEGF, bFGF, and HGF diminish expression of Cav-1, but not of Cav-2, as well as the number of caveolae in human endothelial cells *in vitro* (45). VEGF, PDGF, and HGF also diminish Cav-1 expression in bovine aortic endothelial cells. Cav-1 overexpression in human microvascular endothelial cells leads to increased endothelial cell differentiation and microtubule formation, while inhibition of Cav-1 expression results in a decrease of these structures (48). Thus, Cav-1 participates at distinct steps of the angiogenic process.

Transient overexpression of Cav-1 inhibits VEGF- or serum-induced proliferation of human umbilical vein endothelial cells through inhibition of ERK-1/2 signaling and prevention of VEGF inhibition of p27 and retinoblastoma (Rb) phosphorylation causing an arrest at the G0/G1 phase of the cell cycle (49). Similar results were obtained with ovine fetoplacental artery endothelial cells, in which overexpression of Cav-1 or the use of a CSD peptide, provoked an inhibition of VEGF-stimulated ERK-1/2 activation, cell proliferation, and tube formation. However, VEGF and bFGF do not alter Cav-1 expression (50), as shown in previous studies with human cells (45). Downregulation of endogenous Cav-1 expression with a specific shRNA had the same effect as overexpression of Cav-1, i.e., inhibition of VEGF-induction, ERK-1/2 activation, proliferation and tube formation (50). This may result from the fact that inhibiting endogenous Cav-1 impacts on the generation of caveolae and efficient signaling generated in these membrane domains. Thus, the absence of caveolae would have the same effect on ERK-1/2 activation as overexpression of the inhibitory Cav-1. This apparent paradox was originally explained in the model of Cav-1 inhibition of caveolae-residing endothelial nitric oxide (eNOS), in which overexpression or deletion of Cav-1 caused inhibition of eNOS by either Cav-1-mediated inhibition of eNOS or depletion of caveolae caused by absence of Cav-1, disrupting the appropriate localization of eNOS (51, 52).

*In vivo*, controversial results are reported for the action of Cav-1 and Cav-2 on angiogenesis. Cav-1^−/−^ and Cav-2^−/−^ mice are viable but show many alterations of their phenotypes. In common, these mice show severe pulmonary defects, with hypercellularity, thickening of alveolar septa, and increase in the number of endothelial cells and fibrosis (9, 13). In Cav-2^−/−^ mice lungs, Cav-1 is still expressed but in lower levels (50%). However, in Cav-1^−/−^ mice lungs, the level of Cav-2 is negligible; therefore, the altered lung phenotype of these mice is attributed to the lack of Cav-2, instead of Cav-1.

Lung endothelial cells isolated from Cav-2^−/−^ mice show a higher level of p-ERK-1/2 and p-Rb, express more cyclin A and cyclin B1 and less of the cdk inhibitor p27, all indicative of a high proliferative capacity (53). Accordingly, whole lung lysates from bleomycin-treated Cav-2^−/−^ mice exhibit hypercellularity, express more cyclin D1 and less p27 compared to wild type (54). Thus, the absence of Cav-2 appears to relieve the inhibition of proliferation in these studies. However, this is not the case when isolated endothelial cells are kept in the presence of TGF-β. This factor has an anti-proliferative action on murine lung endothelial cells. Reintroduction of Cav-2 in Cav-2-deficient endothelial cells reduces the inhibitory effect of TGF-β on proliferation to a mild level, similar to the level observed in wild-type cells. Cav-2 counteracts the anti-proliferative role of TGF-β by inhibition of the Alk-5/Smad-2 pathway, with reduced expression of target genes such as plasminogen activator inhibitor-1 and collagen type I. Cav-1, but not Alk-5/Smad-2-3, localizes to caveolae in WT and Cav-2-deficient cells, indicating a Cav-1-independent role of Cav-2 (55). Thus, Cav-2 activity on proliferation of endothelial cells depends on the set of environmental factors.

**Figure 2:**
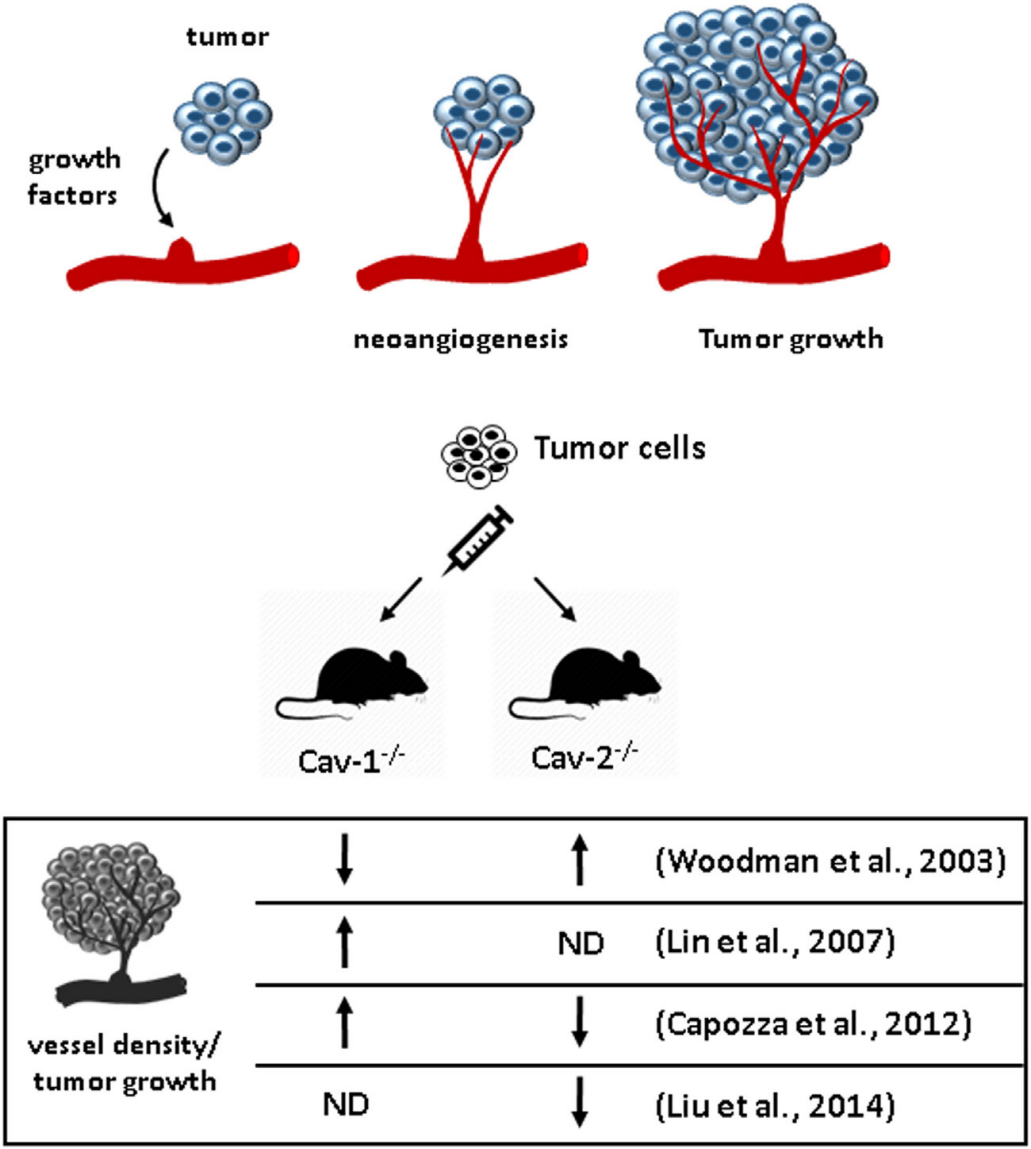
Effect of caveolins in angiogenesis and tumor growth. **(A)** Tumor cells secrete growth factors that stimulate neoangiogenesis toward the tumor. The new vessels supply the tumor with nutrients and support its growth. **(B)** Cav-1^−/−^ and Cav-2^−/−^ mice exhibit opposite effects regarding vessel density and tumor growth after implantation of tumor cells to the skin compared to wild type in two studies that used both mice (56, 57).

Caveolin-1 and Cav-2 show opposite effects in distinct studies of tumor-induced angiogenesis (Figure 2; Table 2). The Lisanti group published two papers reporting controversial results about the ability of Cav-1 and Cav-2 to promote the growth of transplanted B16F10 melanoma cells to the skin of mice (56, 57). In both studies though, the authors found that Cav-1 and Cav-2 affect angiogenesis in opposite ways. In Woodman’s paper, B16F10 tumor cells were transplanted subcutaneously into WT and Cav-1-deficient mice. Cav-1^−/−^ mice showed reduced tumor growth and vessel density. Because the Cav-2 expression is negligible in Cav-1^−/−^ mice, vessel density was also analyzed in Cav-2^−/−^ mice. In contrast to the tumors of Cav-1^−/−^, tumors of Cav-2^−/−^ mice showed an increase in vessel density. Thus, the impairment of tumor-induced angiogenesis in Cav-1^−/−^ mice was attributed to the absence of Cav-1 and not of Cav-2. Surprisingly, in another paper of the same group, Capozza and coworkers injected B16F10 cells intradermally and showed that tumor growth of B16F10 melanoma cells increases in Cav-1^−/−^ mice and decreases in Cav-2^−/−^ mice. They also found that vascular density correlates with tumor growth, being more pronounced in Cav-1-deficient mice and less in Cav-2^−/−^ mice. Further, they demonstrated an important role of fibroblasts in producing factors that stimulate tumor growth and angiogenesis and argue that the reason underlying the distinct results of their work compared to Woodmans’ study may be the microenvironment that surrounds tumor cells, because of the distinct sites of injection (56). There may be other reasons for these findings because two other groups injected tumor cells subcutaneously in Cav-1- and Cav-2^−/−^ mice getting results similar to those from the Capozza study. Sessa’s group injected Lewis lung carcinoma cells in wild-type and Cav-1^−/−^ mice subcutaneously and found that angiogenesis, permeability, and tumor growth were enhanced, effects attributed to an increased phosphorylation of VEGFR and reduced association with VE-cadherin (58). Sowa’s group transplanted melanoma B16F10 cells in Cav-2^−/−^ mice and found that tumor development was strikingly inhibited in the absence of Cav-2. The mechanism of the observed inhibitory effect was attributed to reduced microvascular density associated with an increase in thrombospondin levels and inhibition of Ser1177 phosphorylation of endothelial nitric oxide synthase (59). The discrepancies among the cited studies still await further investigation, but strikingly Cav-1 and Cav-2 seem to act coordinately, with opposing effects.

**Table 2:** Opposing effects of caveolin-1 (Cav-1) and Caveolin-2 (Cav-2) in cellular processes.

Cell process	Cav-1	Cav-2	Reference
Angiogenesis			
Tumor growth and vessel density	Cav-1^−/−^ mice showed reduced B16F10-induced tumor growth and vessel density	B16F10-induced tumors of Cav-2^−/−^ mice show an increase in vessel density	Woodman et al. (57)
	Cav-1^−/−^ mice showed augmented B16F10-induced tumor growth and vessel density	Cav-2^−/−^ mice showed reduced B16F10-induced tumor growth and vessel density	Capozza et al. (56)

Endotoxemia	Cav-1^−/−^ show delayed mortality to endotoxemia, associated with a decrease of activation of STAT-1 and expression of iNOS in intestinal epithelial cells, and do not show alterations in intestinal tissue damage and permeability	Cav-2^−/−^ mice show an increase in susceptibility to endotoxemia associated with an increase in activation of STAT-1, iNOS expression in intestinal epithelial cells, nitric oxide production, intestinal tissue damage, and intestinal permeability	de Almeida et al. (60)

*Salmonella* infection	Cav-1 knockdown diminishes *Salmonella* invasion Cav-1 overexpression increases *Salmonella* invasion	Knockdown of Cav-2 in intestinal epithelial cells increases *Salmonella* invasion	Lim et al. (61) Lim et al. (62) Hoeke et al. (63) Lim et al. (64)

*Pseudomonas aeruginosa* infection	Overexpression of Cav-1 in lung epithelial cells has no effect on *Pseudomonas* invasion	Overexpression of Cav-2 increases bacteria number; knockdown of Cav-2 reduces *Pseudomonas* invasion	Zaas et al. (65)

Fibrosis	Cav-1^−/−^ mice are equally susceptible to bleomycin-induced fibrosis compared to wild-type	Cav-2^−/−^ mice are more susceptible to bleomycin-induced lung fibrosis associated with an increase of apoptosis and proliferation markers compared to wild-type	de Almeida et al. (54)

Insulin-induced proliferation	Cav-1 expression reverses the effects of insulin on ERK-1/2, its translocation to the nucleus, and subsequent increase of cells in S phase	Insulin induces Cav-2 expression, its interaction with phospho-ERK-1/2 that facilitates translocation of ERK-1/2 to the nucleus, and subsequent increase of cells in S phase	Kim and Pak (66)

Endocytosis: mAchR	Ectopic expression of Cav-1 does not alter endocytosis of mAchR, but co-expression with Cav-2 rescues the inhibitory effect of Cav-2 on this process	Ectopic expression of Cav-2 inhibits endocytosis of mAchR	Shmuel et al. (67)

Outflow facility	Cav-1 silencing increases outflow facility	Cav-2 silencing diminishes outflow facility	Aga et al. (68)

*Results of published reports that analyze the activities of both proteins in a same model of study*.

### Infection and Inflammatory Response

Macrophages express both Cav-1 and Cav-2, but these proteins do not co-localize significantly because Cav-1 is mainly present in the plasma membrane and Cav-2 in the Golgi (69). Increasing Cav-1 expression regulates monocyte to macrophage differentiation (70). LPS and other microbial products modulate Cav-1 expression (71, 72), which in macrophages inhibits the production of pro-inflammatory cytokines stimulating the expression of anti-inflammatory ones (24, 25). Interestingly, macrophages derived from primary bone marrow cells only show Cav-2 expression, which is diminished by inflammatory stimuli (73).

**Figure 3:**
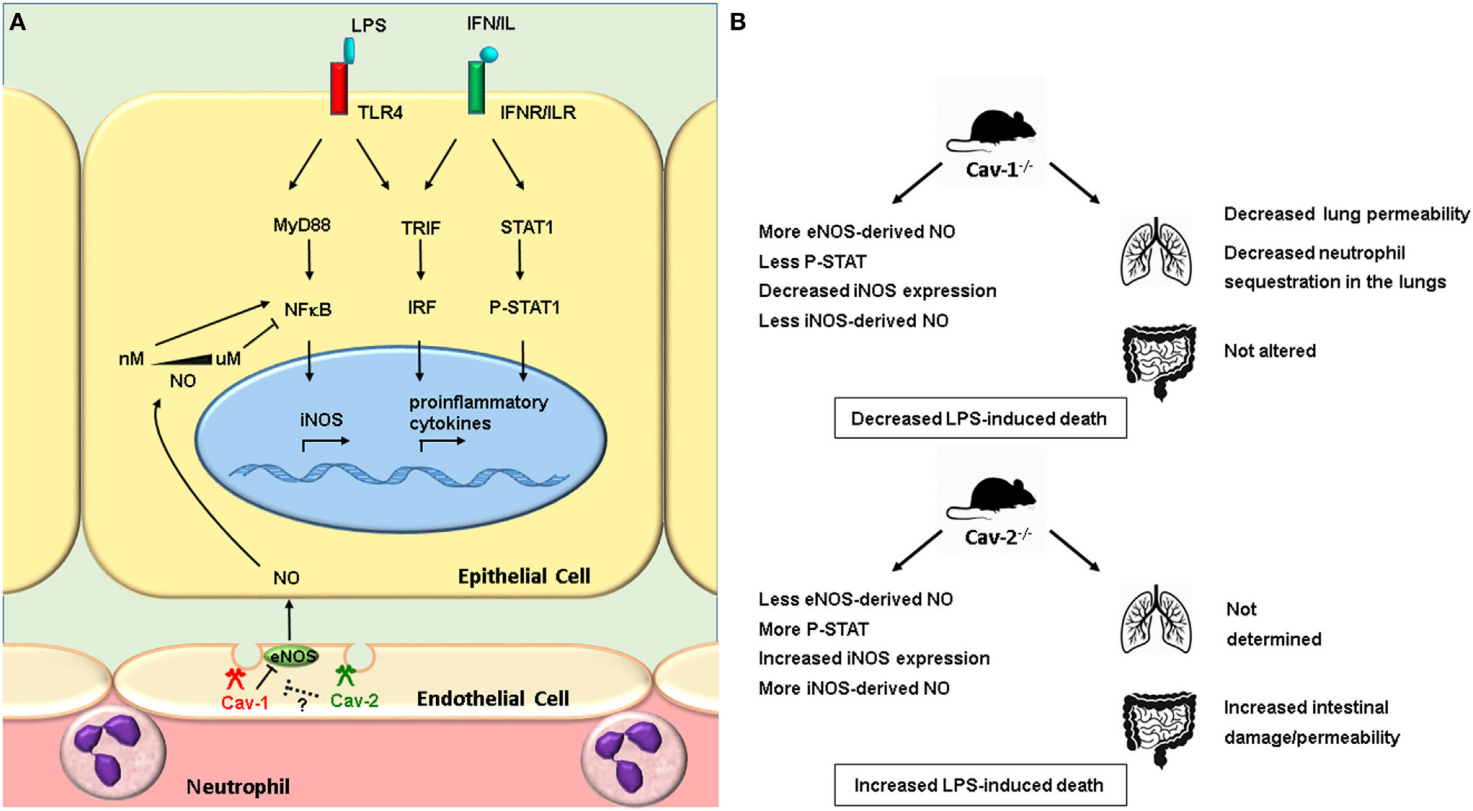
Cav-1^−/−^ and Cav-2^−/−^ mice exhibit opposite outcomes in lipopolysaccharide (LPS)-induced sepsis. LPS binds to the TLR4 receptor and activates MyD88 and TRIF pathways, resulting in activation of the transcription factors NF-κB and IRF, which regulate the transcription of inflammatory cytokines and iNOS. The inflammatory cytokines activate the JAK-STAT pathway, regulating the expression of iNOS. The activation of NF-κB is also regulated by NO. Low concentrations of NO (nanomolar range) activates NF-κB, whereas high concentration (micromolar range) inhibits it (74). In endothelial cells, eNOS resides in caveolae, where it is subjected to inhibition by caveolin-1 (Cav-1). **(B)** LPS-injected Cav-1^−/−^ and Cav-2^−/−^ mice exhibit distinct outcomes of sepsis. Cav-1^−/−^ mice lungs produce higher concentrations of eNOS-derived NO, inhibition of NF-κB, and decreased expression of iNOS compared to wild type (29). The expression of iNOS is also decreased in the gut of Cav-1^−/−^ mice, whereas in the gut of Cav-2^−/−^ mice it is increased compared to wild type. In the lungs of Cav-1^−/−^ mice, the vascular permeability is decreased, less neutrophils are sequestered. On the other hand, the vascular permeability is enhanced in the gut of Cav-2^−/−^ mice (60). See the main text for details.

Cav-1^−/−^ and Cav-2^−/−^ mice helped to elucidate the role of caveolins in the inflammatory response *in vivo*, particularly during sepsis (Figure 3). Cav-1^−/−^ mice are more resistant to LPS-induced death associated with decreased vascular permeability, recruitment of neutrophils and edema formation in the lungs. These effects are attributed to an increase of endothelial-derived NO due to the relieve of eNOS inhibition by Cav-1, and subsequent inhibition of NF-κB activation and nitric oxide synthase (iNOS) transcription (29). Furthermore, the increase of NO in LPS-stimulated lung endothelial cells of Cav-1^−/−^ mice results in nitration of interleukin-1 receptor-associated kinase, loss of kinase activity and impairment of NF-κB activation (28). Accordingly, silencing of Cav-1 gene results in diminished LPS-induced permeability (27).

We have demonstrated that Cav-1 and Cav-2 play important and antagonistic roles in the outcome of sepsis induced by LPS in mice (Table 2). Cav-1^−/−^ mice show delayed mortality after stimulation with LPS, while Cav-2^−/−^ mice are more sensitive. Endotoxin-induced sepsis in Cav-2^−/−^ mice is associated with increased intestinal tissue damage, intestinal permeability, iNOS expression in intestinal epithelial cells, and NO production. In contrast, Cav-1^−/−^ mice show a decrease of iNOS expression and NO production, but no alterations in intestinal permeability. In our hands, Cav-1^−/−^ mice showed no increase in NO production in their peritoneal cavity (60). Differential expression of iNOS is associated with a distinct activation of STAT-1. Intestinal cells of Cav-2^−/−^ show increased phosphorylation of Tyr701 STAT—as compared to wild-type intestinal cells, whereas intestinal cells of Cav-1^−/−^ mice show reduced levels of Tyr701 phosphorylation on STAT-1. In conclusion, Cav-1^−/−^ and Cav-2^−/−^ mice show opposing outcomes in LPS-induced endotoxemia. As Cav-2 is not expressed in Cav-1^−/−^ mice, we conclude that the observed effects in Cav-2^−/−^ mice are not only due to the absence of Cav-2, supporting the idea that the balance of Cav-1 and Cav-2 activities is important for the expression of iNOS and the progress to sepsis (60).

Different from the outcome observed after LPS-induced endotoxemia, Cav-1^−/−^ mice show a higher susceptibility to infections by *Salmonella enterovar*, displaying a higher bacterial burden in the spleen and liver compared to wild-type mice. Interestingly, in isolated macrophages, bacteria uptake is similar for both genotypes. This higher susceptibility is attributed to the higher production of pro-inflammatory cytokines and NO in Cav-1^−/−^ mice, a phenomenon, which is associated with diminished activation of STAT3. These mice also show increased infiltration of neutrophils in *Salmonella*-induced liver granulomas and increased liver necrosis (37). This demonstrates that the role of Cav-1 in the inflammatory response may depend on the inflammatory stimulus that triggered the inflammation and subsequent mechanisms involved.

Other groups also showed that Cav-1 expression correlates to efficient *Salmonella* invasion in non-phagocytic cells. Caco-2 cells (epithelial colorectal adenocarcinoma cells) are devoid of Cav-1 and are not susceptible to *Salmonella*. However, these cells can acquire an M-like phenotype (M-cells are present in the gastrointestinal tract and allow for transport of microbes across the epithelia), expressing high levels of Cav-1, when cocultured with Raji B cells. In this scenario, they become as highly sensitive to *Salmonella* infection as M-cells (61). Senescent cells (BrdU-treated HeLa cells or senescent human diploid fibroblasts) express high levels of Cav-1 and show a higher endocytic activity. Interestingly, these cells are more susceptible to *Salmonella* compared to non-senescent counterparts (62). Accordingly, Cav-1 knockdown inhibits *Salmonella* transcytosis and invasion in M-like cells and senescent cells, while overexpression of Cav-1 in non-senescent cells increases *Salmonella* invasion.

For the invasion of non-phagocytic cells, *Salmonella* counts on the bacterial effector molecules SopE, SopE2, and SopB that are delivered into host cells by the type III secretion system (TTSS) and activate Rho GTPases that regulate cortical actin for the ruffling of the plasma membrane and subsequent bacterial invasion. *Salmonella* activates Rac1 and Cdc42, but only Rac1 is necessary for bacterial invasion through the apical plasma membrane of polarized cells. SopE regulates Rac1 activation in caveolae, which are mobilized to the apical membrane. SopE1 and Rac1 interact with Cav-1, which mediates the activation of Rac1. Conversely, inhibition of Rac1 activation suppresses interaction with Cav-1. Accordingly, Cav-1 knockdown decreases interaction between Rac1 and SopE impairing *Salmonella* invasion (64).

**Figure 4:**
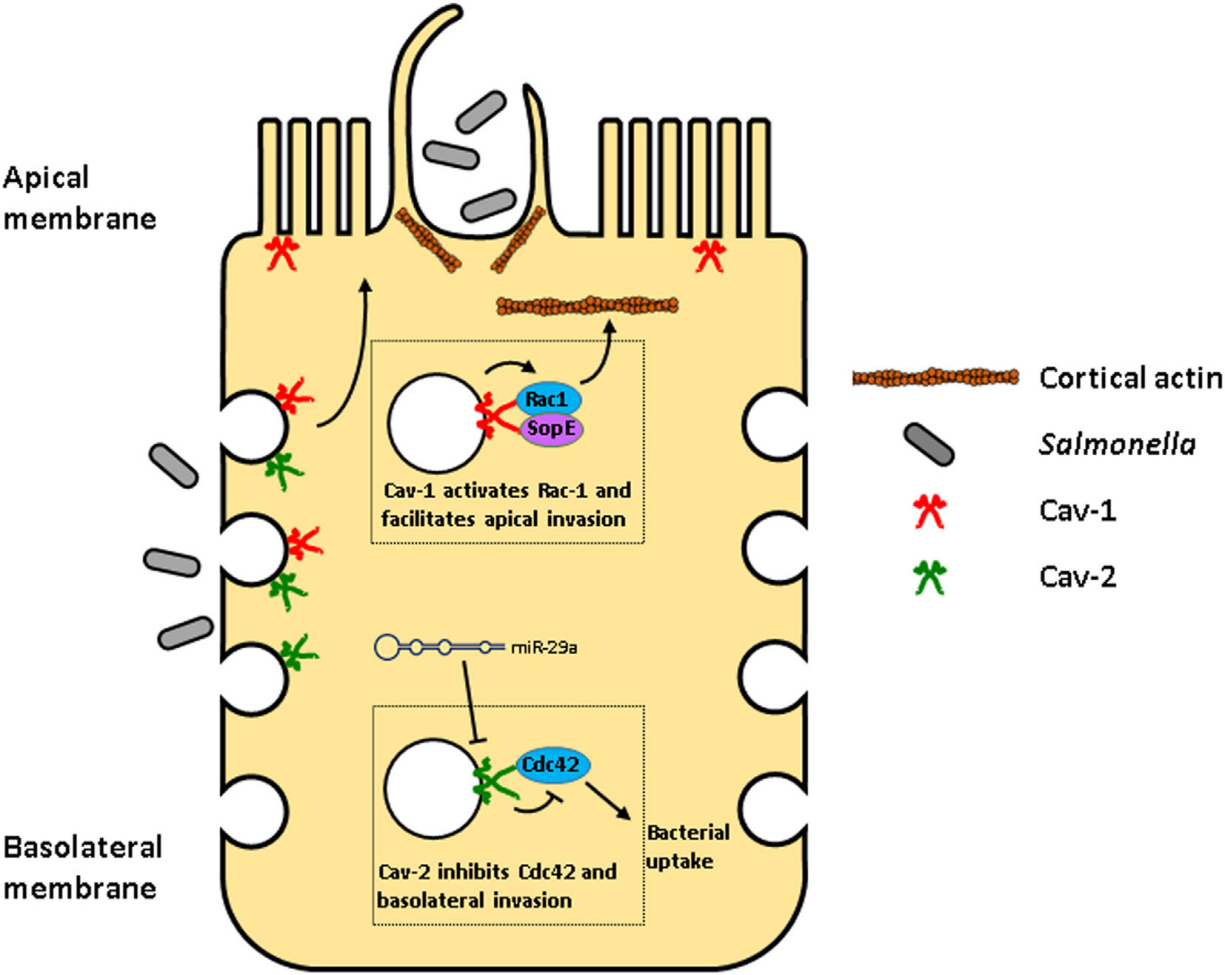
Opposite effects of caveolin-1 (Cav-1) and Caveolin-2 (Cav-2) during *Salmonella* invasion in enterocytes. *Salmonella* can enter enterocytes through the apical plasma membrane or the basolateral membrane using distinct mechanisms. *Salmonella* delivers into host cells, bacterial effector molecules, such as SopE that interacts with Cav-1, mediating the activation of Rac1 in caveolae that are then mobilized to the apical membrane. Activated Rac1 regulates cortical actin resulting in the ruffling of the plasma membrane facilitating bacterial invasion (64). On the other hand, Cav-2 is expressed only in basolateral membranes, where it inhibits Cdc42 activation, decreasing *Salmonella*’s uptake. Interestingly, *Salmonella* induces the transcription of miR-29a, which decreases Cav-2 expression, relieving Cdc42 inhibition, increasing *Salmonella* uptake (63).

Curiously, Cav-2 also impacts *Salmonella* invasion and cell proliferation, but in the opposite way. Unlike Cav-1, Cav-2 is expressed in basolateral membranes, where *Salmonella* is also able to invade the cell, a process that involves activation of Rac1 and Cdc42, but is not dependent on these two GTPases. Cdc42 interacts with SopB and is required for bacterial intracellular replication (75). Piglets infected with *Salmonella* show an increase of miR-29a 3 days post-infection, which correlates with a decreased expression of its targets Cdc42 and Cav-2 both at transcript and protein levels. Transfection of the human intestinal epithelial cell line HT-29 with miR-29a decreases protein levels of Cav-2 as well. Transfection with miR-29a or Cav-2 siRNA does not decrease Cdc42 protein levels in HT-29, but, interestingly, decreases the activation of this protein. Accordingly, these procedures also provoke an increased uptake of *Salmonella* and retarded epithelial proliferation (63). In summary, while Cav-1 facilitates *Salmonella* invasion, Cav-2 inhibits it. Furthermore, Cav-1 is associated with impairment of cell proliferation (47, 76), while Cav-2 promotes it (Figure 4; Table 2).

Caveolin-1 and Cav-2 also show opposing effects regarding infections by the Gram-negative bacteria *Pseudomonas aeruginosa*, although one may observe that disparate results were obtained in *in vivo* studies of *Pseudomonas* infection, as it will be discussed next. These bacteria frequently infect epithelial cells in immunocompromised individuals. Raft disturbing agents impair *Pseudomonas* invasion in lung epithelial cells, but not their adherence. *P. aeruginosa* co-localizes with Cav-1 and Cav-2 and knockdown of Cav-1 or Cav-2 reduces invasion by 50%. Cav-1 silencing results in 60% decrease of Cav-1, but also a significant decrease of Cav-2, whereas Cav-2 silencing results in 80% decrease of Cav-2, but not of Cav-1. Taken together, Cav-2 is the main player in *Pseudomonas* invasion (77). In accordance with this, overexpression of Cav-2 increases bacteria numbers, while overexpression of Cav-1 in lung epithelial cells has no effect on *Pseudomonas* invasion. Cav-2 binds and co-localizes with c-Src and, after *Pseudomonas* invasion, it also associates transiently to Csk at the sites of invasion. Molecular experiments led to the conclusion that invasion of *Pseudomonas* depends on Cav-2 phosphorylation by members of Src family and this process is negatively regulated by Csk. Accordingly, the use of genistein, a phosphotyrosine kinase inhibitor, impairs this microbe infection (65). Besides, lipid raft disruption, which also prevents Cav-2 phosphorylation, suggests that the success of invasion depends both on phosphorylation of Cav-2 and raft integrity (77). Cav-1^−/−^ mice and primary cultures of tracheal epithelial cells derived from these animals are strikingly resistant to *Pseudomonas* infection *in vivo*. The mortality rate is a 100% for the wild-type mice while only half of Cav-1^−/−^ mice die after 48 h, and the survivors recover from the infection. Accordingly, Cav-1^−/−^ mice produce less of the inflammatory cytokines MIP-2, IL-1β, and TNF-α, recruit a lower number of neutrophils, and show decreased lung injury. Interestingly, macrophages of both genotypes internalize *Pseudomonas* equally, indicating the major role of lipid rafts-mediated endocytosis by lung epithelial cells in infection, enabling bacteria to escape phagocytosis by macrophages and replicate (65).

On the other side, Gadjeva and collaborators observed that Cav-1^−/−^ mice are more susceptible compared to wild-type, associated with high bacterial burden in the lung and spleen, and with elevated production of several inflammatory cytokines. Infection is accompanied by neutrophil recruitment in both Cav-1^−/−^ and wild-type, but isolated neutrophils of Cav-1^−/−^ mice are less efficient in phagocytosis. Cav-1^−/−^ mice are colonized more efficiently in a model of chronic infection (78). The authors argue that the contrasting results could be due to the different bacteria strains used or to the path of bacteria delivery (transtracheal instillation vs. inhalation). The same group demonstrated that Cav-1 associates with cystic fibrosis transmembrane conductance regulator and contributes to *Pseudomonas* invasion. They also observed a decrease of *Pseudomonas* invasion after the Cav-1 knockdown in human bronchial epithelial cells (IB-31). The Abraham group obtained a similar result using murine lung epithelial cells (79). A third group working with the same strains and method of infection also observed a higher mortality, lung injury and systemic dissemination of *Pseudomonas* in Cav-1^−/−^ mice compared to wild type. These effects correlate with increased ROS production and increased neutrophil recruitment to the lung. Colonies of bacteria and lipid peroxidation occur in other organs, such as the spleen, liver, and kidneys. Alveolar macrophages show a higher bacterial content in Cav-1^−/−^ compared to wild-type. In culture, isolated macrophages of Cav-1^−/−^ mice phagocyte less than wild-type. The inflammatory response was increased in Cav-1^−/−^ mice compared to wild-type, with regard to detection of inflammatory cytokines in the bronchoalveolar lavage fluid. In the lung tissue, Cav-1^−/−^ mice show a stronger activation of proteins crucial for the inflammatory response, such as NF-κB, STAT3, JAK2, and SOCS3 (80).

Cav-2^−/−^ mice could be used to better understand the effect of Cav-1 and Cav-2 on pathogen infection. Caveolins also play a major role in fibrosis, which could represent a repair process to restore tissue integrity after an acute or chronic inflammation as will be discussed in the next topic.

### Fibrosis

**Figure 5:**
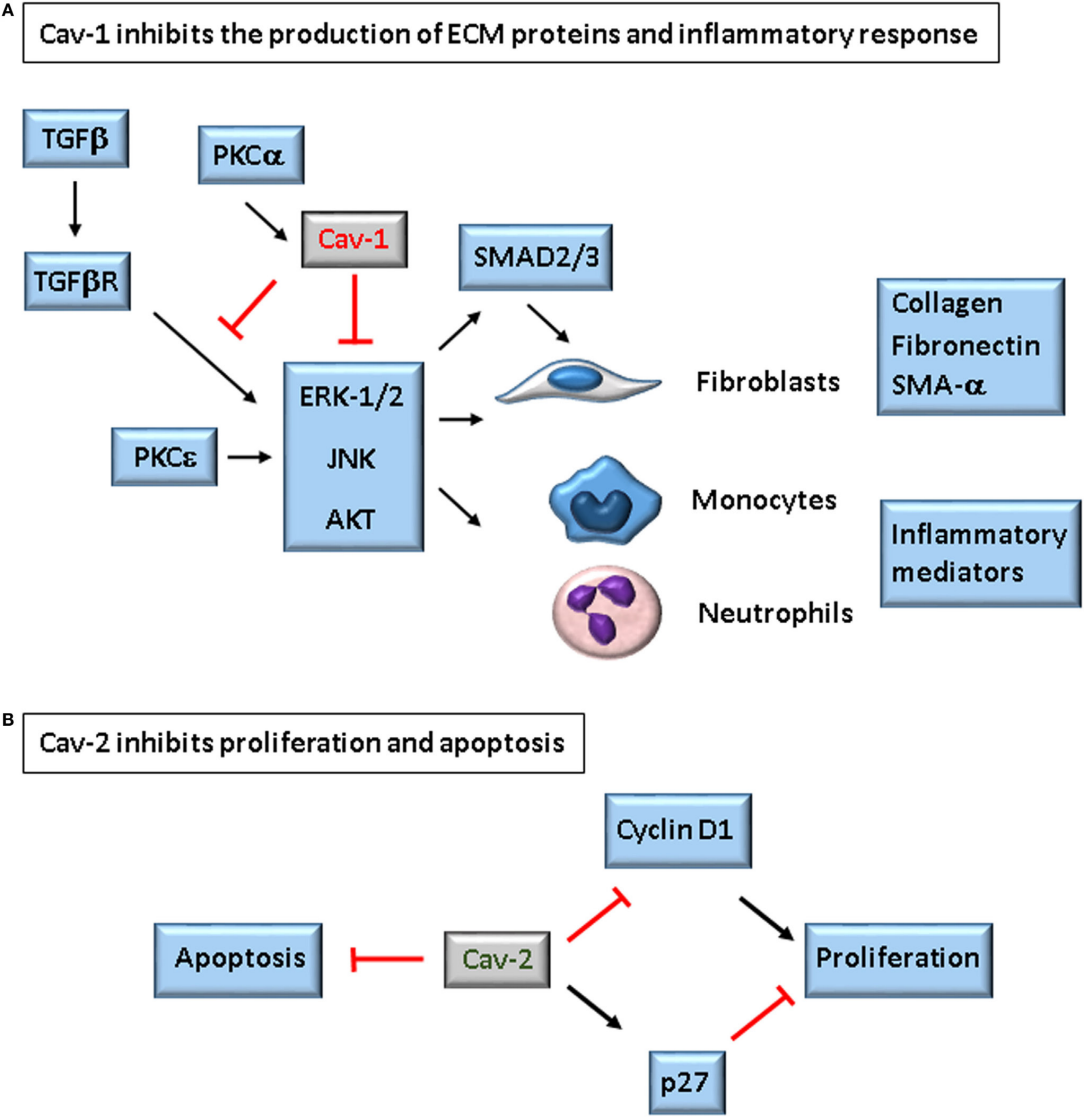
Role of caveolins in the development of fibrosis. Caveolin-1 (Cav-1) inhibits expression of ECM proteins by two described mechanisms: it binds to TGFβ receptor type I (TβR-I), inhibiting Smad-2/3 activation and translocation to the nucleus (81), and/or it inhibits ERK-1/2, JNK, and AKT. In both cases, the result is decreased production of collagen, tenascin, fibronectin, and α-SMA by fibroblasts (40, 42, 43). In leukocytes, Cav-1 restricts the production of inflammatory mediators. On the hand, absence of Caveolin-2 (Cav-2) is associated with increased Cyclin D1 and decreased p27, and increased number of apoptotic cells in the lungs of bleomycin-treated mice. The lungs of Cav-1^−/−^ mice express negligible levels of Cav-2, but these mice do not show increased fibrosis as Cav-2^−/−^ mice, indicating these proteins counterbalance in the development of bleomycin-induced lung fibrosis (60).

Fibrosis is characterized by excessive accumulation of ECM, in particular of collagen. It is a complex process starting with epithelial damage and apoptosis, myofibroblast proliferation and differentiation, and recruitment of inflammatory cells. Cav-1^−/−^ or Cav-2^−/−^mice present thickening of alveolar septa, disorganized parenchyma, hypercellularity, and increased numbers of endothelial cells, all characteristics of fibrosis (9, 13, 14). Both mice suffer from severe pulmonary dysfunction attributed to the absence of Cav-2. However, a substantial literature shows that Cav-1 regulates fibrosis in lung and dermal patient-derived cells and in the mouse model of bleomycin-induced pulmonary fibrosis (40–43). The roles of Cav-1 and Cav-2 in fibrosis will be discussed next, and the main findings of the actions of these proteins are illustrated in Figure 5.

Scleroderma or systemic sclerosis (SSc), an autoimmune disease characterized by the excessive accumulation of collagen in the skin and in internal organs, frequently accompanied by microvascular injury and immunological alteration. Hoffman–Tourkina’s group found that in lung fibroblasts, collagen expression is regulated by PKCε, PKCα, and ERK-1/2. Gain and loss of function experiments revealed that PKCε increases ERK-1/2 activation, whereas PKCα induces Cav-1, which inhibits ERK-1/2. In SSc lung fibroblasts, Cav-1 is reduced, ERK-1/2 is hyperactivated and collagen production is augmented. However, further studies are needed to explain why silencing Cav-1 does not alter ERK-1/2 activation and collagen production, and why ERK-1/2 activation is lower compared to normal lung fibroblasts. Also, SSc dermal fibroblasts show elevated Cav-1 expression, but also possess more activated ERK-1/2 compared to normal dermal fibroblasts. The analysis of bleomycin-injured lungs *in vivo* corroborates that fibrotic tissue is characterized by the lower Cav-1 expression, hyperactivation of ERK-1/2, and excessive collagen production (41).

Wang’s group showed that the lung tissue of idiopathic pulmonary fibrosis patients also have reduced Cav-1 expression both at mRNA and protein levels compared to the lungs of healthy control subjects. The Cav-1 reduction is observed in epithelial and fibroblasts but not in endothelial cells. Furthermore, transfection of Cav-1 diminishes the damage caused by bleomycin in lung tissue of mice, characterized by less fibrosis, disruption of the alveolar unit and inflammatory cell infiltration. These changes are accompanied by a reduction of TGF-β content, Smad-2 phosphorylation, fibronectin accumulation, and collagen production (43). Besides the reduction of TGF-β content, what could explain the reduction of Smad-2 activation, Cav-1 interacts with TGF-β type I receptor through its CSD domain, with subsequent inhibition of Smad-2 phosphorylation and nuclear translocation of Smad-2 (81). Accordingly, gain and loss of function experiments using human primary pulmonary fibroblasts and the human pulmonary fibroblast cell type MRC-5 showed that Cav-1 suppresses the TGF-β signaling pathway, by inhibiting Smad-2 and Smad-3 phosphorylation, their nuclear translocation and thereby the production of SMA-α, collagen, and fibronectin. Furthermore, TGF-β activity decreases Cav-1 expression, while Cav-1 modulates TGF-β-induced collagen and fibronectin production *via* ERK-1/2 and JNK inhibition, respectively (43). Another study showed that overexpression of Cav-1 inhibits production of collagen, tenascin, and SMA-α, whereas Cav-1 silencing has the opposite effect, and that CSD peptide alone has the same effects, both in normal and scleroderma patient-derived fibroblasts. Mechanistically, these effects depend on the inhibitory effect of Cav-1 or CSD peptide on the activity of ERK-1/2, JNK, and Akt (42).

We have also provided evidence that Cav-1 participates of fibrosis in the pathogenesis of SSc. Lungs of SSc and pulmonary arterial hypertension (PAH) patients show decreased expression of Cav-1 in the thickened alveolar septa compared to normal lungs and to non-affected regions of the lungs of the same patients. The skin of SSc patients show reduced levels of Cav-1 compared to normal skin, and so do *in vitro* cultured fibroblasts derived from skin lesions of PAH patients compared to healthy controls. Cav-1^−/−^ mice show enhanced collagen deposition in lungs and skin compared to wild-type. CSD peptide reduces collagen production at basal level and after TGF-β stimulation and significantly diminishes Smad-3 phosphorylation and translocation to the nucleus (40).

Caveolin-1 also has a role in innate immune cells which may contribute to fibrosis. During bleomycin-induced lung fibrosis, leukocytes are recruited to the lungs and secrete inflammatory mediators that damage the tissue and activate fibroblasts. These activated fibroblasts can further differentiate into fibrocytes and, thereby intensify the fibrotic process. Bleomycin treatment also reduces Cav-1 expression in circulating monocytes, as well as in monocytes and neutrophils found in the lung tissue, compared to saline. This effect is associated with hyperactivation of MAPK (ERK, JNK, and p38) signaling pathways, which potentially contributes to an exaggerated inflammatory response. Monocytes, polymorphonuclear cells, and T cells (but not B cells) of SSc patients also show diminished Cav-1 expression and enhanced expression of p-ERK-1/2, p-JNK, and p-p38 and of inflammatory markers, such as Cox-2 and CXCR4. Treatment with CSD abrogates leukocyte recruitment and reverses MAPK hyperactivation, CXCR4 and MMP9 overexpression in monocytes of either normal or SSc origin, as well as in TGF-β-treated monocytes (82). These results corroborate the studies that implicate Cav-1 as a modulator of the inflammatory response, as aforementioned in this review. SSc monocytes express high levels of CXCR4 and migrate to CXCL12 faster, and this effect is reversible by CSD. Also TGF-β-treated fibroblasts express lower levels of Cav-1, higher levels of CXCR4 and migrate more effectively, adding more evidence for the role of Cav-1 in fibrosis *via* modulation of TGF-β signaling (83). Cav-1 may be involved in the predisposition African-Americans show to develop interstitial lung disease in patients with SSc. Monocytes from African-Americans express less Cav-1 and migrate more efficiently toward the CXCR4-ligand SDF-1 compared to Caucasian-derived monocytes (84). Monocytes from African-Americans also express more chemokine receptors (CCRs), show increased Src activation and increased migration toward MCP-1 and MCP-3. All these alterations are reversible by CSD treatment (85).

In summary, many evidence points toward an anti-fibrotic property of Cav-1. The mechanisms involved include inhibition of MAPK and Src kinases activation, inhibition of TGF-β signaling pathway, inhibition of expression of inflammatory mediators of cell activation and cell migration. Further, Cav-1 influences the fibrosis outcome acting on distinct cell types, such as epithelial and endothelial cells, fibroblasts and leukocytes.

Although the altered morphology of lungs in Cav-1 and Cav-2-deficient mice was attributed to the absence of Cav-2 (9), Cav-2 was not directly investigated as a potential agent in fibrosis-like Cav-1. We showed that Cav-2 is indeed involved by different mechanisms. Treatment of wild-type mice with bleomycin drastically reduces the expression of the beta isoform of Cav-2 in the lung and its phosphorylation at Tyr19 and Ser23. Further studies are necessary to understand the importance of Cav-2 phosphorylation in fibrosis though. Cav-2 phosphorylation at Ser23 and Ser36 has been implicated in the formation of deep caveolae (18), phosphorylation of Cav-2 at Tyr19 disrupts interaction with Cav-1 (19) and is required for *P. aeruginosa* infection (77). Phosphorylation of Tyr19 and Tyr27 occur upon EGF stimulation at distinct time points and cause differential subcellular localization (20). Cav-2 and its beta isoform are found in lipid droplets (86) and mouse lung fibroblasts can be classified in two distinct subpopulations. One class is characterized as Thy1^+^, spindle-shaped cells, rich in lipid droplets and able to secrete high amounts of collagen, but not fibronectin. The other class is Thy-1^−^ round-shaped cells without lipid droplets, and with a higher capacity to secrete fibronectin than collagen (87). Is the beta isoform of Cav-2 specifically involved in the regulation of a fibrosis-prone profile of fibroblasts? Distinct Cav-1 isoforms are associated with specific cell types of the lung. For instance, alveolar type I epithelial cells mainly express the β isoform of Cav-1, whereas endothelial cells are rich in the Cav-1 α isoform (88). The Cav-1 α-isoform, but not the β-isoform is implicated with the formation of deep caveolae (17). There is—to my best knowledge—no report about Cav-2 isoforms distribution in the lung. The striking decrease observed in the β isoform of Cav-2, however, may well correspond to a direct effect in specific cell types.

Cav-2^−/−^ mice, but not Cav-1^−/−^ mice are more susceptible to bleomycin-induced damage compared to wild-type (Table 2). The injury is characterized by alveolar thickening, increased cell density, and deposition of collagen. Interestingly, the exacerbated effect of bleomycin in Cav-2^−/−^ mice compared to Cav-1^−/−^ and wild-type mice does not seem to be associated with the TGF-β signaling pathway because there is no alteration in TβRI expression or in the activation state of Smad-2/3 in the different genotypes. Accordingly, although Cav-2^−/−^lungs contain more collagen, their ability to produce collagen is not altered, based on the variation observed between the collagen content of lungs of saline-instilled and bleomycin-treated mice in each genotype. Apoptosis and proliferation though are more prominent in the lungs of bleomycin-treated Cav-2^−/−^ mice compared to wild type. We could not identify which cell type was apoptotic in our studies, but apoptosis of epithelial cells is an important trigger of lung fibrotic response (89), and an expressive increase of apoptosis may underlie the exacerbated bleomycin-induced injury observed in Cav-2^−/−^ mice compared to wild type. The investigation if phosphorylation of Cav-2 is involved in apoptosis of epithelial cells will provide further important information here.

Fibrosis is accompanied by proliferation and accumulation of mesenchymal cells in the lung parenchyma. Endothelial cells also proliferate during neovascularization of fibrotic foci (89). The role of Cav-1 and Cav-2 in endothelial proliferation and differentiation during fibrosis requires further investigation, because both apparently interfere with tumor-induced angiogenesis in distinct ways, as discussed before. Bleomycin-injured lungs of Cav-2^−/−^ mice show increased expression of Cyclin D1 and decreased expression of p27; an increase of Cyclin A and B1, and a decrease of p27 were also observed in mouse lung endothelial cells (53). Because Cav-1^−/−^ mice express negligible amounts of Cav-2 and respond differently to treatment with bleomycin, we conclude that Cav-1 and Cav-2 have distinct roles in bleomycin-induced fibrosis and the balance between the two Cav proteins determines the development of fibrotic process (54).

## Antagonistic Actions of Cav-1 and Cav-2 in Other Cellular Processes

### Insulin-Induced Proliferation

Caveolin-1 expression induces cell cycle arrest. The mechanisms include repression of Cyclin D1 transcription and p53/p21 activation (46, 47). One of the diverse actions of ERK-1/2 on cell cycle control involves both Cav-1 and Cav-2 with antagonistic activities (Table 2) (66). Hirc-B cells are Rat-1-derived fibroblasts that overexpress the insulin receptor. In these cells, Cav-2 is the major caveolin and Cav-1 is expressed at reduced levels. Insulin treatment of Hirc-B cells induces Cav-2 expression and stimulates the phosphorylation of ERK-1/2, as well as the interaction of p-ERK-1/2 with Cav-2, translocation of p-ERK-1/2 into the nucleus and a sixfold induction of the number of cells in S phase. Transfection of Cav-1 reverts this process (66). The interaction of Cav-2 with p-ERK-1/2 depends on phosphorylation of Cav-2 on Tyr19. Silencing of Cav-2 impairs not ERK-1/2 activation, but the insulin-induced nuclear translocation of p-ERK-1/2, with subsequent reduction of expression of c-Jun, cyclin D1, and DNA synthesis (90).

As mentioned in the Section “Fibrosis,” the correlation of Cav-2 and cyclin D1 was distinct to what we found in the lungs of bleomycin-instilled mice, where we observed a striking reduction of Cav-2 phosphorylation at Tyr19 and Ser23, as well as an enhancement of cyclin D1 expression, although we did not access which lung cells were directly affected. Cav-2^−/−^ mice lungs also showed a further increase in expression of cyclin D1 (54).

### Endocytosis

As for other cellular processes mentioned above, the role of caveolins in endocytosis is also controversial. The morphology of caveolae suggests a role in the concentration and internalization of substances. It is the case for the SV40 virus or cholera toxin B, that preferentially enters the cell through caveolae, by interaction with ganglioside GM1, although they can also use alternative pathways (91, 92). Nevertheless, some characteristics of caveolae also argue against a general role in endocytosis (93): (1) unlike clathrin-coated vesicles, caveolae are not ubiquitous; (2) mice deficient in caveolins are viable, while deletion or silencing of clathrin results in slow growth and embryonic lethality in a variety of animals (94); (3) only 5% of all caveolae are endocytosed and these structures exclude bulk proteins (95–97). Therefore, caveolae are not ideal candidates for an essential role in overall endocytosis. Caveolae might rather participate in the internalization of specific cargos. It also has been suggested that caveolae are involved with lipid metabolism (97), and might serve as a membrane reservoir protecting the plasma membrane against mechanical stress (93, 98).

Though the role of caveolae in endocytosis is still a matter of debate, Cav-1 is able to inhibit endocytosis through several distinct caveolae- or clathrin-dependent and independent mechanisms (92, 99, 100). Besides Cav-1, cavin-1, and cavin-3 also inhibit the CLIC–GEEC pathway, a clathrin-independent endocytic pathway, distinct from the caveolar one (99).

So far, only two studies, using distinct models, report opposing activities of Cav-1 and Cav-2 in endocytosis (Table 2) (67, 68). Schmuel and collaborators investigated the regulation of clathrin-mediated endocytosis of the M1 muscarinic receptor (mAChR) by caveolins. They found that Cav-1 and Cav-2 act in concert and that the balance of the expression of both proteins controls mAchR internalization. This receptor resides on the basolateral membrane of MDCK cells and is endocytosed upon stimulation with agonists, such as carbachol. Ectopic expression of Cav-1 does not alter mAChR endocytosis, whereas expression of Cav-2 inhibits it dramatically. Interestingly, co-expression with Cav-1 rescues the inhibitory effect of Cav-2. mAChR co-localizes with Cav-2 in intracellular compartments in cells expressing Cav-2 alone, but localizes to the plasma membrane and intracellular compartments when Cav-1 and Cav-2 are co-expressed. The authors propose a model in which its interaction with Cav-2 impairs clathrin-coated pits mediated endocytosis of mAChR. Cav-1 dependent sequestration of Cav-2 would then disrupt Cav-2 inhibitory effect (67).

In two further studies, caveolins were implicated in the control of intraocular pressure (IOP) (68, 101). The authors propose endocytosis and mechano-protection as mechanisms. IOP is the main risk factor for glaucoma and is regulated by the conventional outflow pathway. This consists of the trabecular meshwork (TM) and the Schlemm’s canal (SC) that controls the drainage of the aqueous humor of the eye, and consequently, IOP. In *ex vivo* perfused organ cultures of both human and porcine anterior segments, Cav-1 silencing facilitates outflow, whereas Cav-2 silencing has the opposite effect (68). The authors suggest that IOP is modulated by the turnover of ECM protein in a process involving caveolin-regulated endocytosis. Cav-1 and Cav-2 partially co-localize in TM, and both co-localize with cortactin, an archetypal marker of podosome- or invadopodia-like structures (PILS) (68). These structures are rich in matrix metalloproteinases (MMPs) and are involved in focal degradation and turnover of ECM. The addition of MMPs in the perfusion medium of anterior chambers increases outflow (102). MMP2, MMP14, and ADAMTS4 co-localize, to different degrees, with Cav-1 and Cav-2. Interestingly, silencing of Cav-1 or Cav-2 leads to enhanced expression of MMP2, MMP14, and ADAMTS4 increased degradation of gelatin and increased fibronectin in TM tissue and fibrillar fibronectin in TM cells. The antagonizing effects, which silencing of Cav-1 or Cav-2 cause on IOP, do not correlate with effects on ECM turnover though and the cause of the effects on IOP requires further investigation (68).

On the other hand, other studies show that Cav-1^−/−^ mice exhibit ocular hypertension and reduced outflow in apparent disagreement with Aga’s study (101, 103, 104). Elliott and collaborators argue that in Aga’s study, the silencing of Cav-1 may not have been complete and that the remaining protein may be responsible for the contrasting results obtained after complete removal of Cav-1. Cav-1 KO mice express negligible amounts of Cav-2 because Cav-2 expression strictly depends on Cav-1 (see above). Partial silencing of Cav-1 though results in some Cav-2 expression. Efficient Cav-2 silencing likewise results in a strong reduction of Cav-2, but maintenance of Cav-1 expression. It is possible, that in this system, the number and quality of caveolae after Cav-1 or Cav-2 silencing may be altered, but this awaits further studies. In Cav-1^−/−^ mice, eNOS is hyperactivated, probably due to Cav-1 inhibition relief, and partially rescues the outflow deficiency (101, 104). Moreover, variations of the ocular pulse can be studied by cyclic mechanical stretching of TM cells. TM and SC are rich in caveolae, where Cav-1 and Cav-2 co-localize and where partial co-localization of cavin-1 is detected. Mechanical stimulation causes cavin dissociation from caveolins, indicating a disassembly of caveolae. Cav-1^−/−^ SC show higher membrane damage after induction of high IOP. Hence, the authors propose that Cav-1 has a mechano-protection role in the eye (101). Interestingly, polymorphisms at CAV1/2 loci are associated both with glaucoma and with IOP (101).

## Conclusion and Perspectives

In this review, we summarized the literature that considers Cav-1 and Cav-2 as antagonists for the regulation of several cellular processes such as endothelial proliferation, endocytosis, infection, inflammatory response, and fibrosis. Cav-1 and Cav-2 are co-expressed in most cells, except striated muscle cells, and they generally co-localize in caveolae. Cav-1 is a necessary constituent of caveolae and Cav-2 is able to modulate certain characteristics of these membrane structures. Many proteins are concentrated in caveolae, which function as cellular platforms for appropriate signal transduction. Some of these proteins bind to and are regulated by Cav-1 such as eNOS, EGFR, and Src kinases. Besides their activity in caveolae, caveolins are found in other cellular compartments like Golgi, mitochondria, nucleus, and lipid droplets, where they participate in the regulation of a variety of cellular processes. These include gene expression, protein glycosylation, protection of mitochondrial function, and lipid transport (105) (Table 1). It is still unclear, whether or not specific activities of Cav-1 and Cav-2 depend on their localization into caveolae. Some caution is recommended when analyzing results obtained by artificially eliminating or overexpressing caveolins as these may result in the ablation of caveolae. In cases where the subcellular localization of caveolins is altered, such as what happens after Cav-1 overexpression, this mislocalization may cause effects on its own, beyond those caused by the expression level alone. In the absence of Cav-1, no caveolae are formed and Cav-2 localizes to different subcellular locations if it is expressed at all. In some studies, cells naturally devoid of Cav-1 and Cav-2 are used to ectopically express one or both of these proteins. Though these cells may be useful tools to study the role of each caveolin independently, they also may not completely reflect the real functions of caveolins expressed in cells that naturally express caveolins.

Despite the intrinsic problems of some strategies used so far to study caveolin function, a variety of results obtained in different models indicate that Cav-1 and Cav-2, frequently acting in concert and seen as partners, may indeed cause antagonistic effects (Tables 1 and 2). To our knowledge, this is the first review that specifically addresses this issue. Until today, the amount of work dedicated to Cav-2 and its activities is small compared to the large knowledge accumulated on Cav-1. Future work about these proteins working in concert may elucidate how the regulation of cellular processes is affected by the different caveolin family members.

## Author Contributions

CA is the only author of this review, being responsible for the conception and drafting of it.

## Conflict of Interest Statement

The author declares that the research was conducted in the absence of any commercial or financial relationships that could be construed as a potential conflict of interest.

## References

[1] Couet J Li S Okamoto T Ikezu T Lisanti MP Identification of peptide and protein ligands for the caveolin-scaffolding domain. Biochemistry (1997) 272:6525–33. 10.1074/jbc.272.10.6525 9045678

[2] Okamoto T Schlegel A Scherer PE Lisanti MP Caveolins, a family of scaffolding proteins for organizing “preassembled signaling complexes” at the plasma membrane. J Biol Chem (1998) 273:5419–22. 10.1074/jbc.273.10.5419 9488658

[3] Yamamoto M Toya Y Schwencke C Lisanti MP Myers MG Jr Ishikawa Y. Caveolin is an activator of insulin receptor signaling. J Biol Chem (1998) 273:26962–8. 10.1074/jbc.273.41.26962 9756945

[4] Byrne DP Dart C Rigden DJ. Evaluating caveolin interactions: do proteins interact with the caveolin scaffolding domain through a widespread aromatic residue-rich motif? PLoS One (2012) 7:e44879. 10.1371/journal.pone.0044879 23028656 PMC3444507

[5] Collins BM Davis MJ Hancock JF Parton RG. Structure-based reassessment of the caveolin signaling model: do caveolae regulate signaling through caveolin-protein interactions? Dev Cell (2012) 23:11–20. 10.1016/j.devcel.2012.06.012 22814599 PMC3427029

[6] Scherer PE Lewis Y Volonte D Engelman JA Galbiati F Couet J Cell-type and tissue-specific expression of caveolin-2. J Biol Chem (1997) 272:29337–46. 10.1074/jbc.272.46.29337 9361015

[7] Liu L Pilch PF A critical role of cavin (polymerase I and transcript release factor) in caveolae formation and organization. J Biol Chem (2008) 283:4314–22. 10.1074/jbc.M707890200 18056712

[8] Li S Song K Lisanti M Expression and characterization of recombinant caveolin. J Biol Chem (1996) 271:568–73. 10.1074/jbc.271.1.568 8550621

[9] Razani B Wang XB Engelman JA Lagaud G Zhang XL Kneitz B Caveolin-2-deficient mice show evidence of severe pulmonary dysfunction without disruption of caveolae caveolin-2-deficient mice show evidence of severe pulmonary dysfunction without disruption of caveolae. Mol Cell Biol (2002) 22:2329–44. 10.1128/MCB.22.7.2329 11884617 PMC133690

[10] Li S Galbiati F Volonte D Sargiacomo M Das K Scherer PE Mutational analysis of caveolin-induced vesicle formation. FEBS Lett (1998) 434:127–34. 10.1016/S0014-5793(98)00945-4 9738464

[11] Mora R Bonilha VL Marmorstein A Scherer PE Brown D Lisanti MP Caveolin-2 localizes to the Golgi complex but redistributes to plasma membrane, caveolae, and rafts when co-expressed with caveolin-1. J Biol Chem (1999) 274:25708–17. 10.1074/jbc.274.36.25708 10464308

[12] Parolini I Sargiacomo M Galbiati F Rizzo G Grignani F Engelman JA Expression of caveolin-l is required for the transport caveolin-2 to the plasma membrane. Mol Biol Cell (1999) 10:313A. 10464309 10.1074/jbc.274.36.25718

[13] Drab M Verkade P Elger M Kasper M Lohn M Lauterbach B Loss of caveolae, vascular dysfunction, and pulmonary defects in caveolin-1 gene-disrupted mice. Science (2001) 293:2449–52. 10.1126/science.1062688 11498544

[14] Razani B Engelman JA Wang XB Schubert W Zhang XL Marks CB Caveolin-1 null mice are viable but show evidence of hyperproliferative and vascular abnormalities. J Biol Chem (2001) 276:38121–38. 10.1074/jbc.M105408200 11457855

[15] Hu G Ye RD Dinauer MC Malik AB Minshall RD Neutrophil caveolin-1 expression contributes to mechanism of lung inflammation and injury. Am J Physiol Lung Cell Mol Physiol (2008) 294:L178–86. 10.1152/ajplung.00263.2007 17993589

[16] Lahtinen U Honsho M Parton RG Simons K Verkade P. Involvement of caveolin-2 in caveolar biogenesis in MDCK cells. FEBS Lett (2003) 538:85–8. 10.1016/S0014-5793(03)00135-2 12633858

[17] Fujimoto T Kogo H Nomura R Une T. Isoforms of caveolin-1 and caveolar structure. J Cell Sci (2000) 113(Pt 19):3509–17. 10984441 10.1242/jcs.113.19.3509

[18] Sowa G Pypaert M Fulton D Sessa WC. The phosphorylation of caveolin-2 on serines 23 and 36 modulates caveolin-1-dependent caveolae formation. Proc Natl Acad Sci U S A (2003) 100:6511–6. 10.1073/pnas.1031672100 12743374 PMC164477

[19] Lee H Park DS Wang XB Scherer PE Schwartz PE Lisanti MP Src-induced phosphorylation of caveolin-2 on tyrosine 19. Phospho-caveolin-2 (Tyr(P)19) is localized near focal adhesions, remains associated with lipid rafts/caveolae, but no longer forms a high molecular mass hetero-oligomer with caveolin-1. J Biol Chem (2002) 277:34556–67. 10.1074/jbc.M204367200 12091389

[20] Wang XB Lee H Capozza F Marmon S Sotgia F Brooks JW Tyrosine phosphorylation of caveolin-2 at residue 27: differences in the spatial and temporal behavior of phospho-Cav-2 (pY19 and pY27). Biochemistry (2004) 43:13694–706. 10.1021/bi049295+ 15504032

[21] Mercier I Jasmin J Pavlides S Minetti C Flomenberg N Pestell RG Clinical and translational implications of the caveolin gene family: lessons from mouse models and human genetic disorders. Lab Invest (2009) 89:614–23. 10.1038/labinvest.2009.23 19333235 PMC2796209

[22] Jiao H Zhang Y Yan Z Wang Z-G Liu G Minshall RD Caveolin-1 Tyr14 phosphorylation induces interaction with TLR4 in endothelial cells and mediates MyD88-dependent signaling and sepsis-induced lung inflammation. J Immunol (2013) 191:6191–9. 10.4049/jimmunol.1300873 24244013 PMC3874812

[23] Lim JS Nguyen KCT Nguyen CT Jang IS Han JM Fabian C Flagellin-dependent TLR5/caveolin-1 as a promising immune activator in immunosenescence. Aging Cell (2015) 14:907–15. 10.1111/acel.12383 26223660 PMC4568978

[24] Wang XM Kim HP Nakahira K Ryter SW Choi AMK. The heme oxygenase-1/carbon monoxide pathway suppresses TLR4 signaling by regulating the interaction of TLR4 with caveolin-1. J Immunol (2009) 182:3809–18. 10.4049/jimmunol.0712437 19265160

[25] Wang XM Kim HP Song R Choi AMK Caveolin-1 confers antiinflammatory effects in murine macrophages via the MKK3/p38 MAPK pathway. Am J Respir Cell Mol Biol (2006) 34:434–42. 10.1165/rcmb.2005-0376OC 16357362 PMC2644205

[26] Lee C-Y Lai T-Y Tsai M-K Chang Y-C Ho Y-H Yu I-S The ubiquitin ligase ZNRF1 promotes caveolin-1 ubiquitination and degradation to modulate inflammation. Nat Commun (2017) 8:15502. 10.1038/ncomms15502 28593998 PMC5472178

[27] Tiruppathi C Shimizu J Miyawaki-Shimizu K Vogel SM Bair AM Minshall RD Role of NF-κB-dependent caveolin-1 expression in the mechanism of increased endothelial permeability induced by lipopolysaccharide. J Biol Chem (2008) 283:4210–8. 10.1074/jbc.M703153200 18077459 PMC2435298

[28] Mirza MK Yuan J Gao X-P Garrean S Brovkovych V Malik AB Caveolin-1 deficiency dampens toll-like receptor 4 signaling through eNOS activation. Am J Pathol (2010) 176:2344–51. 10.2353/ajpath.2010.091088 20304961 PMC2861099

[29] Garrean S Gao X-P Brovkovych V Shimizu J Zhao Y-Y Vogel SM Caveolin-1 regulates NF-kappaB activation and lung inflammatory response to sepsis induced by lipopolysaccharide. J Immunol (2006) 177:4853–60. 10.4049/jimmunol.177.7.4853 16982927

[30] Jiang R Cai J Zhu Z Chen D Wang J Wang Q Hypoxic trophoblast HMGB1 induces endothelial cell hyperpermeability via the TRL-4/caveolin-1 pathway. J Immunol (2014) 193:5000–12. 10.4049/jimmunol.1303445 25339669

[31] Sun Y Hu G Zhang X Minshall RD. Phosphorylation of caveolin-1 regulates oxidant-induced pulmonary vascular permeability via paracellular and transcellular pathways. Circ Res (2009) 105:676–85. 10.1161/CIRCRESAHA.109.201673 19713536 PMC2776728

[32] Marmon S Hinchey J Oh P Cammer M de Almeida CJ Gunther L Caveolin-1 expression determines the route of neutrophil extravasation through skin microvasculature. Am J Pathol (2009) 174:684–92. 10.2353/ajpath.2009.080091 19164603 PMC2630575

[33] Millán J Hewlett L Glyn M Toomre D Clark P Ridley AJ. Lymphocyte transcellular migration occurs through recruitment of endothelial ICAM-1 to caveola- and F-actin-rich domains. Nat Cell Biol (2006) 8:113–23. 10.1038/ncb1356 16429128

[34] Hu G Ye RD Dinauer MC Malik AB Minshall RD. Neutrophil caveolin-1 expression contributes to mechanism of lung inflammation and injury. Am J Physiol Lung Cell Mol Physiol (2008) 294:L178–86. 10.1152/ajplung.00263.2007 17993589

[35] Li J Scherl A Medina F Frank PG Kitsis RN Tanowitz HB Impaired phagocytosis in caveolin-1 deficient macrophages. Cell Cycle (2005) 4:1599–607. 10.4161/cc.4.11.2117 16205123

[36] Tsai T-H Chen S-F Huang T-Y Tzeng C-F Chiang A-S Kou YR Impaired Cd14 and Cd36 expression, bacterial clearance, and toll-like receptor 4-Myd88 signaling in caveolin-1-deleted macrophages and mice. Shock (2011) 35:92–9. 10.1097/SHK.0b013e3181ea45ca 20601931

[37] Medina FA De Almeida CJ Dew E Li J Bonuccelli G Williams TM Caveolin-1-deficient mice show defects in innate immunity and inflammatory immune response during *Salmonella enterica* serovar typhimurium infection. Infect Immun (2006) 74:6665–74. 10.1128/IAI.00949-06 16982844 PMC1698056

[38] Machado FS Rodriguez NE Adesse D Garzoni LR Esper L Lisanti MP Recent developments in the interactions between caveolin and pathogens. Adv Exp Med Biol (2012) 729:65–82. 10.1007/978-1-4614-1222-9 22411314 PMC3564053

[39] Rosenberger CM Brumell JH Finlay BB. Microbial pathogenesis: lipid rafts as pathogen portals. Curr Biol (2000) 10:823–5. 10.1016/S0960-9822(00)00788-0 11102822

[40] Del Galdo F Sotgia F de Almeida CJ Jasmin J-F Musick M Lisanti MP Decreased expression of caveolin 1 in patients with systemic sclerosis: crucial role in the pathogenesis of tissue fibrosis. Arthritis Rheum (2008) 58:2854–65. 10.1002/art.23791 18759267 PMC2770094

[41] Tourkina E Gooz P Pannu J Bonner M Scholz D Hacker S Opposing effects of protein kinase Calpha and protein kinase Cepsilon on collagen expression by human lung fibroblasts are mediated via MEK/ERK and caveolin-1 signaling. J Biol Chem (2005) 280:13879–87. 10.1074/jbc.M412551200 15691837

[42] Tourkina E Richard M Gööz P Bonner M Pannu J Harley R Antifibrotic properties of caveolin-1 scaffolding domain in vitro and in vivo. Am J Physiol Lung Cell Mol Physiol (2008) 294:L843–61. 10.1152/ajplung.00295.2007 18203815

[43] Wang XM Zhang Y Kim HP Zhou Z Feghali-Bostwick CA Liu F Caveolin-1: a critical regulator of lung fibrosis in idiopathic pulmonary fibrosis. J Exp Med (2006) 203:2895–906. 10.1084/jem.20061536 17178917 PMC1850940

[44] Scherer PE Lisanti MP Baldini G Sargiacomo M Mastick CC Lodish HF. Induction of caveolin during adipogenesis and association of GLUT4 with caveolin-rich vesicles. J Cell Biol (1994) 127:1233–43. 10.1083/jcb.127.5.1233 7962086 PMC2120260

[45] Liu J Razani B Tang S Terman BI Ware JA Lisanti MP Angiogenesis activators and inhibitors differentially regulate caveolin-1 expression and caveolae formation in vascular endothelial cells. J Biol Chem (1999) 274:15781–5. 10.1074/jbc.274.22.15781 10336480

[46] Galbiati F Volonté D Liu J Capozza F Frank PG Zhu L Caveolin-1 expression negatively regulates cell cycle progression by inducing G(0)/G(1) arrest via a p53/p21(WAF1/Cip1)-dependent mechanism. Mol Biol Cell (2001) 12:2229–44. 10.1091/mbc.12.8.2229 11514613 PMC58591

[47] Hulit J Bash T Fu M Galbiati F Albanese C Sage DR The cyclin D1 gene is transcriptionally repressed by caveolin-1. J Biol Chem (2000) 275:21203–9. 10.1074/jbc.M000321200 10747899

[48] Liu J Wang XB Park DS Lisanti MP. Caveolin-1 expression enhances endothelial capillary tubule formation. J Biol Chem (2002) 277:10661–8. 10.1074/jbc.M110354200 11748236

[49] Fang K Fu W Beardsley AR Sun X Lisanti MP Liu J. Overexpression of caveolin-1 inhibits endothelial cell proliferation by arresting the cell cycle at G0/G1 phase. Cell Cycle (2007) 6:199–204. 10.4161/cc.6.2.3740 17245131

[50] Liao W-X Feng L Zhang H Zheng J Moore TR Chen D-B. Compartmentalizing VEGF-induced ERK2/1 signaling in placental artery endothelial cell caveolae: a paradoxical role of caveolin-1 in placental angiogenesis in vitro. Mol Endocrinol (2009) 23:1428–44. 10.1210/me.2008-0475 19477952 PMC2737550

[51] Feron O Kelly RA The caveolar paradox: suppressing, inducing, and terminating eNOS signaling. Circ Res (2001) 88:129–31. 10.1161/01.RES.88.2.129 11157661

[52] Sbaa E Frérart F Feron O. The double regulation of endothelial nitric oxide synthase by caveolae and caveolin: a paradox solved through the study of angiogenesis. Trends Cardiovasc Med (2005) 15:157–62. 10.1016/j.tcm.2005.05.006 16165011

[53] Xie L Frank PG Lisanti MP Sowa G. Endothelial cells isolated from caveolin-2 knockout mice display higher proliferation rate and cell cycle progression relative to their wild-type counterparts. Am J Physiol Cell Physiol (2010) 298:C693–701. 10.1152/ajpcell.00401.2009 20007452 PMC2838569

[54] de Almeida CJG Jasmin JF Del Galdo F Lisanti MP. Genetic ablation of caveolin-2 sensitizes mice to bleomycin-induced injury. Cell Cycle (2013) 12:2248–54. 10.4161/cc.25335 24067367 PMC3755075

[55] Xie L Vo-Ransdell C Abel B Willoughby C Jang S Sowa G Caveolin-2 is a negative regulator of anti-proliferative function and signaling of transforming growth factor beta in endothelial cells. Am J Physiol Cell Physiol (2011) 301(5):C1161–74. 10.1152/ajpcell.00486.2010 21832243 PMC3213920

[56] Capozza F Trimmer C Castello-Cros R Katiyar S Whitaker-Menezes D Follenzi A Genetic ablation of Cav1 differentially affects melanoma tumor growth and metastasis in mice: role of Cav1 in Shh heterotypic signaling and transendothelial migration. Cancer Res (2012) 72:2262–74. 10.1158/0008-5472.CAN-11-2593 22396494 PMC3342428

[57] Woodman SE Ashton AW Schubert W Lee H Williams TM Medina FA Caveolin-1 knockout mice show an impaired angiogenic response to exogenous stimuli. Am J Pathol (2003) 162:2059–68. 10.1016/S0002-9440(10)64337-4 12759260 PMC1868145

[58] Lin MI Yu J Murata T Sessa WC Caveolin-1-deficient mice have increased tumor microvascular permeability, angiogenesis, and growth. Cancer Res (2007) 67:2849–56. 10.1158/0008-5472.CAN-06-4082 17363608

[59] Liu Y Jang S Xie L Sowa G. Host deficiency in caveolin-2 inhibits lung carcinoma tumor growth by impairing tumor angiogenesis. Cancer Res (2014) 74:6452–62. 10.1158/0008-5472.CAN-14-1408 25269481 PMC4233177

[60] de Almeida CJ Witkiewicz AK Jasmin JF Tanowitz HB Sotgia F Frank PG Caveolin-2-deficient mice show increased sensitivity to endotoxemia. Cell Cycle (2011) 10:2151–61. 10.4161/cc.10.13.16234 21670588 PMC3154364

[61] Lim JS Na HS Lee HC Choy HE Park SC Han JM Caveolae-mediated entry of *Salmonella typhimurium* in a human M-cell model. Biochem Biophys Res Commun (2009) 390:1322–7. 10.1016/j.bbrc.2009.10.145 19879241

[62] Lim JS Choy HE Park SC Han JM Jang IS Cho KA. Caveolae-mediated entry of *Salmonella typhimurium* into senescent nonphagocytotic host cells. Aging Cell (2010) 9:243–51. 10.1111/j.1474-9726.2010.00554.x 20096033 PMC2848979

[63] Hoeke L Sharbati J Pawar K Keller A Einspanier R Sharbati S. Intestinal *Salmonella typhimurium* infection leads to miR-29a induced caveolin 2 regulation. PLoS One (2013) 8(6):e67300. 10.1371/journal.pone.0067300 23826261 PMC3691122

[64] Lim JS Shin M Kim HJ Kim KS Choy HE Cho KA. Caveolin-1 mediates *Salmonella* invasion via the regulation of SopE-dependent Rac1 activation and actin reorganization. J Infect Dis (2014) 210:793–802. 10.1093/infdis/jiu152 24625804

[65] Zaas DW Swan ZD Brown BJ Li G Randell SH Degan S Counteracting signaling activities in lipid rafts associated with the invasion of lung epithelial cells by *Pseudomonas aeruginosa*. J Biol Chem (2009) 284(15):9955–64. 10.1074/jbc.M808629200 19211560 PMC2665119

[66] Kim S Pak Y. Caveolin-2 regulation of the cell cycle in response to insulin in Hirc-B fibroblast cells. Biochem Biophys Res Commun (2005) 330:88–96. 10.1016/j.bbrc.2005.02.130 15781236

[67] Shmuel M Nodel-Berner E Hyman T Rouvinski A Altschuler Y. Caveolin 2 regulates endocytosis and trafficking of the M1 muscarinic receptor in MDCK epithelial cells. Mol Biol Cell (2007) 18:1570–85. 10.1091/mbc.E06-07-0618 17314410 PMC1855036

[68] Aga M Bradley JM Wanchu R Yang Y-F Acott TS Keller KE Differential effects of caveolin-1 and -2 knockdown on aqueous outflow and altered extracellular matrix turnover in caveolin-silenced trabecular meshwork cells. Invest Opthalmol Vis Sci (2014) 55:5497 10.1167/iovs.14-14519 PMC4152152 25103269

[69] Gargalovic P Dory L. Caveolin-1 and caveolin-2 expression in mouse macrophages: high density lipoprotein 3-stimulated secretion and a lack of significant subcellular co-localization. J Biol Chem (2001) 276:26164–70. 10.1074/jbc.M011291200 11316799

[70] Fu Y Moore XL Lee MKS Fernández-Rojo MA Parat MO Parton RG Caveolin-1 plays a critical role in the differentiation of monocytes into macrophages. Arterioscler Thromb Vasc Biol (2012) 32:117–25. 10.1161/ATVBAHA.112.254151 22772753

[71] Lei MG Tan X Qureshi N Morrison DC. Regulation of cellular caveolin-1 protein expression in murine macrophages by microbial products. Society (2005) 73:8136–43. 10.1128/IAI.73.12.8136 16299308 PMC1307083

[72] Lei MG Morrison DC. Differential expression of caveolin-1 in lipopolysaccharide-activated murine macrophages. Infect Immun (2000) 68:5084–9. 10.1128/IAI.68.9.5084-5089.2000 10948129 PMC101744

[73] Maceckova M Martiskova H Koudelka A Kubala L Lojek A Pekarova M. Bone marrow-derived macrophages exclusively expressed caveolin-2: the role of inflammatory activators and hypoxia. Immunobiology (2015) 220:1266–74. 10.1016/j.imbio.2015.06.018 26215374

[74] Connelly I Palacios-Callender M Amexia C Moncada S Hobbs AJ Biphasic regulation of NF-kB activity underlies the pro- and anti-inflammatory actions of nitric oxide. J Immunol (2001) 166:3873–81. 10.4049/jimmunol.166.6.3873 11238631

[75] Rodríguez-Escudero I Ferrer NL Rotger R Cid VJ Molina M. Interaction of the *Salmonella typhimurium* effector protein SopB with host cell Cdc42 is involved in intracellular replication. Mol Microbiol (2011) 80:1220–40. 10.1111/j.1365-2958.2011.07639.x 21435037

[76] Galbiati F Engelman JA Volonte D Zhang XL Minetti C Li M Caveolin-3 null mice show a loss of caveolae, changes in the microdomain distribution of the dystrophin-glycoprotein complex, and T-tubule abnormalities. J Biol Chem (2001) 276:21425–33. 10.1074/jbc.M100828200 11259414

[77] Zaas DW Duncan MJ Li G Wright JR Abraham SN. *Pseudomonas* invasion of type I pneumocytes is dependent on the expression and phosphorylation of caveolin-2. J Biol Chem (2005) 280(6):4864–72. 10.1074/jbc.M411702200 15545264

[78] Gadjeva M Paradis-Bleau C Priebe GP Fichorova R Pier GB. Caveolin-1 modifies the immunity to *Pseudomonas aeruginosa*. J Immunol (2010) 184:296–302. 10.4049/jimmunol.0900604 19949109 PMC2900931

[79] Bajmoczi M Gadjeva M Alper SL Pier GB Golan DE Cystic fibrosis transmembrane conductance regulator and caveolin-1 regulate epithelial cell internalization of *Pseudomonas aeruginosa*. Am J Physiol Cell Physiol (2009) 297:C263–77. 10.1152/ajpcell.00527.2008 19386787 PMC2724095

[80] Yuan K Huang C Fox J Gaid M Weaver A Li G Elevated inflammatory response in caveolin-1-deficient mice with *Pseudomonas aeruginosa* infection is mediated by STAT3 protein and nuclear factor κB (NF-κB). J Biol Chem (2011) 286:21814–25. 10.1074/jbc.M111.237628 21515682 PMC3122236

[81] Razani B Zhang XL Bitzer M Von Gersdorff G Böttinger EP Lisanti MP Caveolin-1 regulates transforming growth factor (TGF)-beta/SMAD signaling through an interaction with the TGF-beta type I receptor. J Biol Chem (2001) 276:6727–38. 10.1074/jbc.M008340200 11102446

[82] Tourkina E Richard M Oates J Hofbauer A Bonner M Gööz P Caveolin-1 regulates leucocyte behaviour in fibrotic lung disease. Ann Rheum Dis (2010) 69:1220–6. 10.1136/ard.2009.117580 20410070 PMC2907085

[83] Tourkina E Bonner M Oates J Hofbauer A Richard M Znoyko S Altered monocyte and fibrocyte phenotype and function in scleroderma interstitial lung disease: reversal by caveolin-1 scaffolding domain peptide. Fibrogenesis Tissue Repair (2011) 4:15. 10.1186/1755-1536-4-15 21722364 PMC3155832

[84] Reese C Perry B Heywood J Bonner M Visconti RP Lee R Caveolin-1 deficiency may predispose African Americans to systemic sclerosis-related interstitial lung disease. Arthritis Rheumatol (2014) 66:1909–19. 10.1002/art.38572 24578173 PMC4158912

[85] Lee R Reese C Perry B Heywood J Bonner M Zemskova M Enhanced chemokine-receptor expression, function, and signaling in healthy African American and scleroderma-patient monocytes are regulated by caveolin-1. Fibrogenesis Tissue Repair (2015) 8:11. 10.1186/s13069-015-0028-7 26322128 PMC4551709

[86] Fujimoto T Kogo H Ishiguro K Tauchi K Nomura R Caveolin-2 is targeted to lipid droplets, a new “membrane domain” in the cell. J Cell Biol (2001) 152:1079–85. 10.1083/jcb.152.5.1079 11238462 PMC2198803

[87] Penney DP Keng PC Derdak S Phipps RP. Morphologic and functional characteristics of subpopulations of murine lung fibroblasts grown in vitro. Anat Rec (1992) 232:432–43. 10.1002/ar.1092320312 1543267

[88] Kogo H Aiba T Fujimoto T Cell type-specific occurrence of caveolin-1 and -1 in the lung caused by expression of distinct mRNAs. J Biol Chem (2004) 279:25574–81. 10.1074/jbc.M310807200 15067006

[89] Uhal BD Apoptosis in lung fibrosis and repair. Chest (2002) 122:293–8. 10.1378/chest.122.6_suppl.293S 12475803

[90] Kwon H Jeong K Pak Y. Identification of pY19-caveolin-2 as a positive regulator of insulin-stimulated actin cytoskeleton-dependent mitogenesis. J Cell Mol Med (2009) 13:1549–64. 10.1111/j.1582-4934.2009.00391.x 19778377 PMC3828866

[91] Damm EM Pelkmans L Kartenbeck J Mezzacasa A Kurzchalia T Helenius A. Clathrin- and caveolin-1-independent endocytosis: entry of simian virus 40 into cells devoid of caveolae. J Cell Biol (2005) 168:477–88. 10.1083/jcb.200407113 15668298 PMC2171728

[92] Lajoie P Kojic LD Nim S Li L Dennis JW Nabi IR. Caveolin-1 regulation of dynamin-dependent, raft-mediated endocytosis of cholera toxin-B sub-unit occurs independently of caveolae. J Cell Mol Med (2009) 13:3218–25. 10.1111/j.1582-4934.2009.00732.x 19438805 PMC4516479

[93] Cheng JPX Nichols BJ. Caveolae: one function or many? Trends Cell Biol (2016) 26(3):177–89. 10.1016/j.tcb.2015.10.010 26653791

[94] Royle SJ The cellular functions of clathrin. Cell Mol Life Sci (2006) 63:1823–32. 10.1007/s00018-005-5587-0 16699812 PMC3475309

[95] Bitsikas V Corrêa IR Nichols BJ Clathrin-independent pathways do not contribute significantly to endocytic flux. Elife (2014) 3:e03970 10.7554/eLife.03970 25232658 PMC4185422

[96] Pelkmans L Zerial M. Kinase-regulated quantal assemblies and kiss-and-run recycling of caveolae. Nature (2005) 436:128–33. 10.1038/nature03866 16001074

[97] Shvets E Bitsikas V Howard G Hansen CG Nichols BJ. Dynamic caveolae exclude bulk membrane proteins and are required for sorting of excess glycosphingolipids. Nat Commun (2015) 6:6867. 10.1038/ncomms7867 25897946 PMC4410672

[98] Parton RG del Pozo MA. Caveolae as plasma membrane sensors, protectors and organizers. Nat Rev Mol Cell Biol (2013) 14:98–112. 10.1038/nrm3512 23340574

[99] Chaudhary N Gomez GA Howes MT Lo HP McMahon KA Rae JA Endocytic crosstalk: cavins, caveolins, and caveolae regulate clathrin-independent endocytosis. PLoS Biol (2014) 12:e1001832 10.1371/journal.pbio.1001832 24714042 PMC3979662

[100] Le PU Guay G Altschuler Y Nabi IR. Caveolin-1 is a negative regulator of caveolae-mediated endocytosis to the endoplasmic reticulum. J Biol Chem (2002) 277:3371–9. 10.1074/jbc.M111240200 11724808

[101] Elliott MH Ashpole NE Gu X Herrnberger L McClellan ME Griffith GL Caveolin-1 modulates intraocular pressure: implications for caveolae mechanoprotection in glaucoma. Sci Rep (2016) 6:37127. 10.1038/srep37127 27841369 PMC5107904

[102] Bradley JMB Vranka J Colvis CM Conger DM Alexander JP Fisk AS Effect of matrix metalloproteinases activity on outflow in perfused human organ culture. Invest Opthalmol Vis Sci (1998) 39:2649–58. 9856774

[103] Kizhatil K Chlebowski A Tolman NG Freeburg NF Ryan MM Shaw NN An in vitro perfusion system to enhance outflow studies in mouse eyes. Invest Opthalmol Vis Sci (2016) 57:5207–15. 10.1167/iovs.16-19481 27701632 PMC5054733

[104] Lei Y Song M Wu J Xing C Sun X. eNOS activity in CAV1 knockout mouse eyes. Invest Opthalmol Vis Sci (2016) 57:2805–13. 10.1167/iovs.15-18841 27228562

[105] Fridolfsson HN Roth DM Insel PA Patel HH. Regulation of intracellular signaling and function by caveolin. FASEB J (2014) 28:3823–31. 10.1096/fj.14-252320 24858278 PMC4139902

